# The Horrors of Sanitation

**Published:** 1923-08

**Authors:** 


### THE HORRORS OF SANITATION.

WE are so accustomed to regard the sanitary engineer as a bulwark against infectious diseases that we are apt to be shocked when he is portrayed as an insufferable nuisance. In a lecture recently delivered in Denmark, Dr. M. Hindhede, the distinguished vegetarian, gave an entertaining account of visits he had lately paid to garden cities in England and Germany. The villain of the piece in England would seem to be the sewer-building sanitarian, whose costly schemes not only rob the land of much useful manure, but add greatly to the cost of living. Having to pay much for their drains, the occupants of garden cities cluster together, so that the same system of drains may serve several houses. The result of this tendency is that garden cities come more and more to resemble the ugly town barracks from which, and because of which, their inhabitants are supposed to have fled. Dr. Hindhede pictures the sanitary engineer as a sort of mechanical fiend pursuing into the country the "simple-lifer" who has fled from him and his abominations in a town. When he visited the colony of Eden, near Berlin, Dr. Hindhede found it blessed—dare we say cursed?—with a total absence of drains and sewers. Water was laid on, but all the sewage was returned to the soil, and the cabbages grown thereon bloomed, if they did not smell, like a rose. But, indeed, there were no smells—at least no evil smells—in this modern Eden, and in support of his praise of the colony Dr. Hindhede stated that its infant death-rate during the first year of life for the past twenty years was only 3.8 per cent. The death-rate for the same age at Letchworth was 5.5 per cent. These and other statistics are quoted in support of the proposition that the sanitary engineer is a meddlesome creature who, if he could, would steal all our valuable waste products and pile theft on theft by sending in a bill for his act of larceny. Though his arguments may not be convincing, Dr. Hindhede's views deserve serious as well as amused consideration.

